# Pericardial Effusion as the First Presentation of Systemic Lupus Erythematosus in a 22-Month-Old Infant: A Case Report and Literature Review

**DOI:** 10.7759/cureus.80042

**Published:** 2025-03-04

**Authors:** Anood Al Rawahi, Saif Awlad Thani, Mohammed Alriyami, Abdullah Al Furqani, Safiya Al-abrawi

**Affiliations:** 1 Pediatrics, Oman Medical Specialty Board, Muscat, OMN; 2 Pediatric Critical Care, The Royal Hospital, Muscat, OMN; 3 Pediatric Nephrology, The Royal Hospital, Muscat, OMN; 4 Pediatric Cardiology, The Royal Hospital, Muscat, OMN; 5 Pediatric Rheumatology, The Royal Hospital, Muscat, OMN

**Keywords:** immunosuppressive therapy, infant, lupus nephritis, pericardial effusion, systemic lupus erythematosus (sle)

## Abstract

Childhood-onset systemic lupus erythematosus (cSLE) is a multi-systemic, inflammatory autoimmune disease that affects many organs including the heart. Pericardial effusion as a primary manifestation of SLE in early infancy is very rare. It has been reported as the first symptom of SLE in adult and adolescent case reports only and the youngest reported case was a three-year-old. We report a case of a 22-month-old infant who had previously been healthy but presented with pericardial effusion and a reduced ejection fraction of 20%. She progressed to cardiogenic shock and acute renal failure and required invasive ventilation, inotropic support and temporary dialysis. She was diagnosed with SLE that was genetically confirmed as autosomal recessive SLE. Her condition improved significantly after starting SLE management with immunosuppression therapy. Pericardial effusion has resolved with medical therapy only and cardiac dysfunction has recovered. According to the available literature, this is the youngest reported case of SLE manifesting as pericardial effusion. This case highlights the importance of including SLE in the differential diagnosis for infants presenting with pericarditis, myocarditis, or pericardial effusion to guide early intervention and reduce risks associated with the disease.

## Introduction

Systemic lupus erythematosus is a multi-systemic, inflammatory autoimmune disease that affects many organs including the heart [1,2]. Childhood-onset systemic lupus erythematosus (cSLE) is a term used for children less than 18 years old. It is a rare disease with an incidence of 0.3-0.9 per 100,000 children per year and a prevalence of 3.3-8.8 per 100,000 children [2]. There are several clinical manifestations associated with SLE, including prolonged fever, rash, arthritis, arthralgia, myalgia, anemia, and thrombocytopenia [3]. SLE can cause cardiac involvement, including pericarditis, myocarditis, endocarditis and pericardial effusions [4]. Pericardial effusion has been reported as the first sign of SLE in adults and adolescents, but has not been reported in children under the age of three years [5-8]. This is the first report of an infant with pericardial effusion as the first manifestation of SLE.

## Case presentation

**Figure 1:**
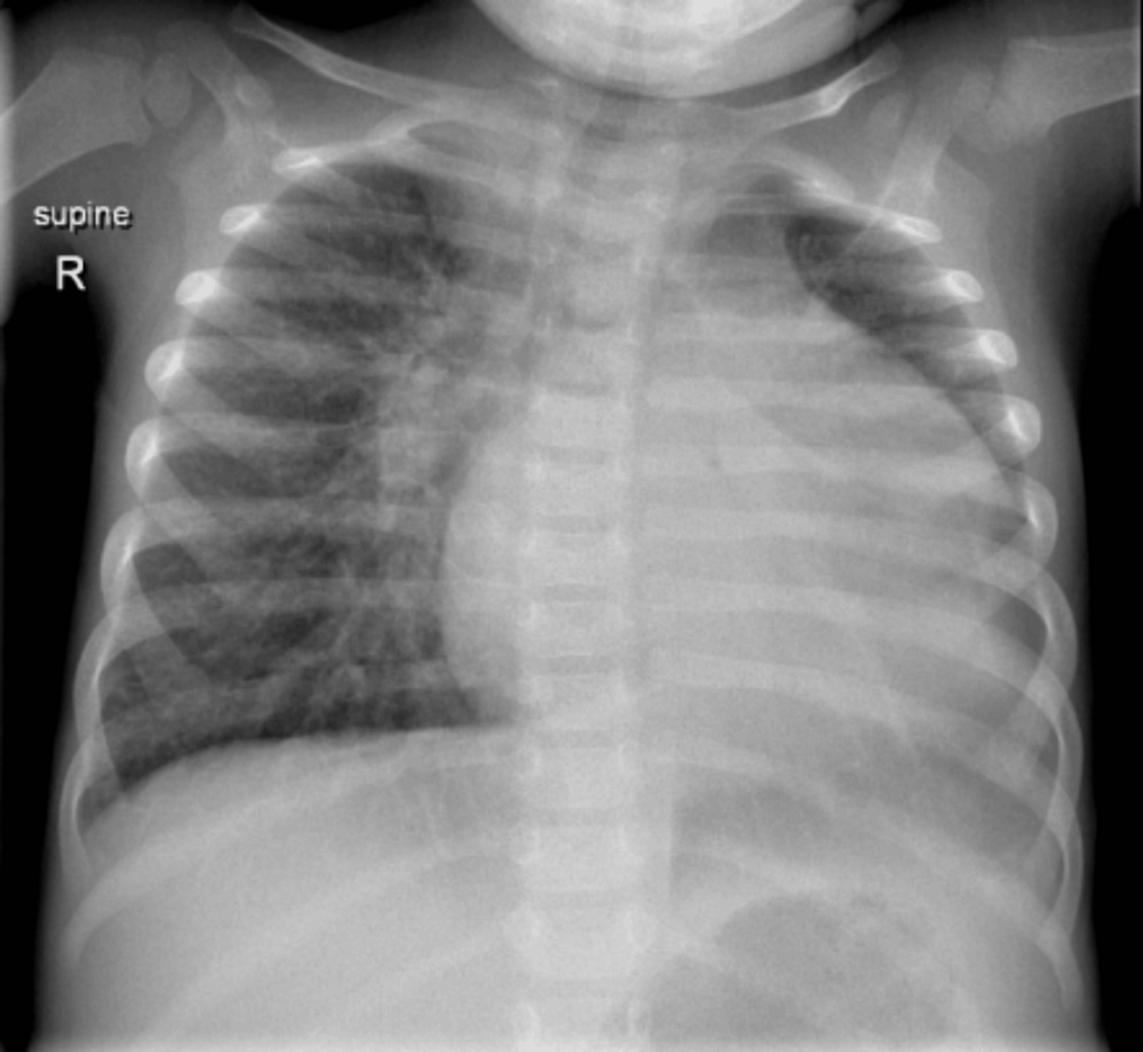
At presentation the chest X-ray shows cardiomegaly

An otherwise healthy 22-month-old female, whose parents are first-degree relatives and no history of maternal SLE, presented to the hospital with swelling of her periorbital region, abdomen, and legs. On examination, she was afebrile (temperature: 36.3 °C), tachycardic (heart rate: 140 beats per minute), tachypnic (respiratory rate: 45 breaths per minute), and hypertensive (blood pressure: 120/88). The chest examination showed subcoastal and suprasternal retraction with good air entry, and the cardiac examination revealed muffled heart sounds and gallop rhythm. The abdomen was soft but distended with ascites, and the liver was four cm below the costal margin. The chest X-ray revealed cardiomegaly (Figure 1).

**Figure 2:**
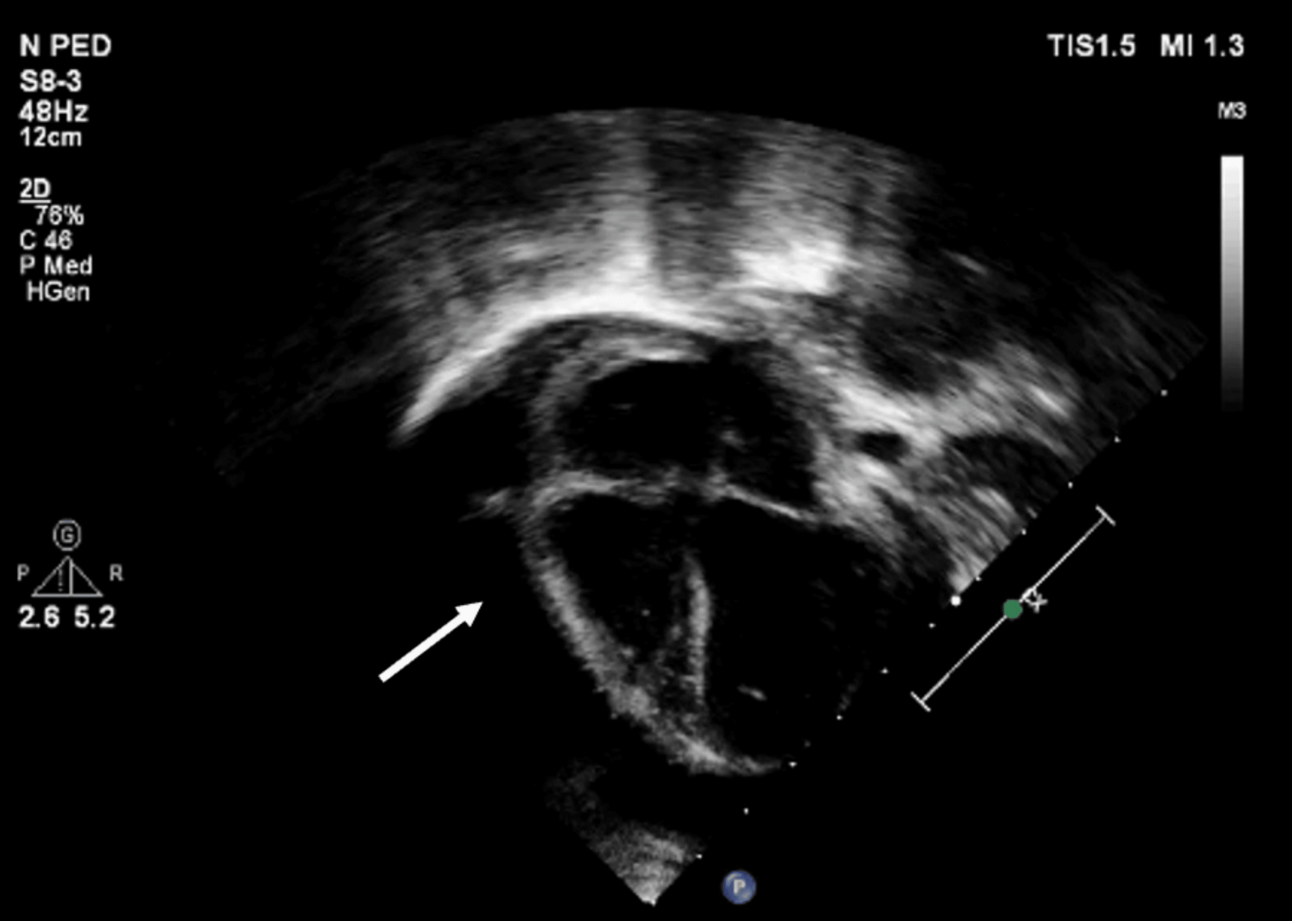
Transthoracic echocardiography (TTE) apical four chamber and parasternal short axis 2D view showing circumferential large pericardial effusion (at the time of presentation)

**Figure 3:**
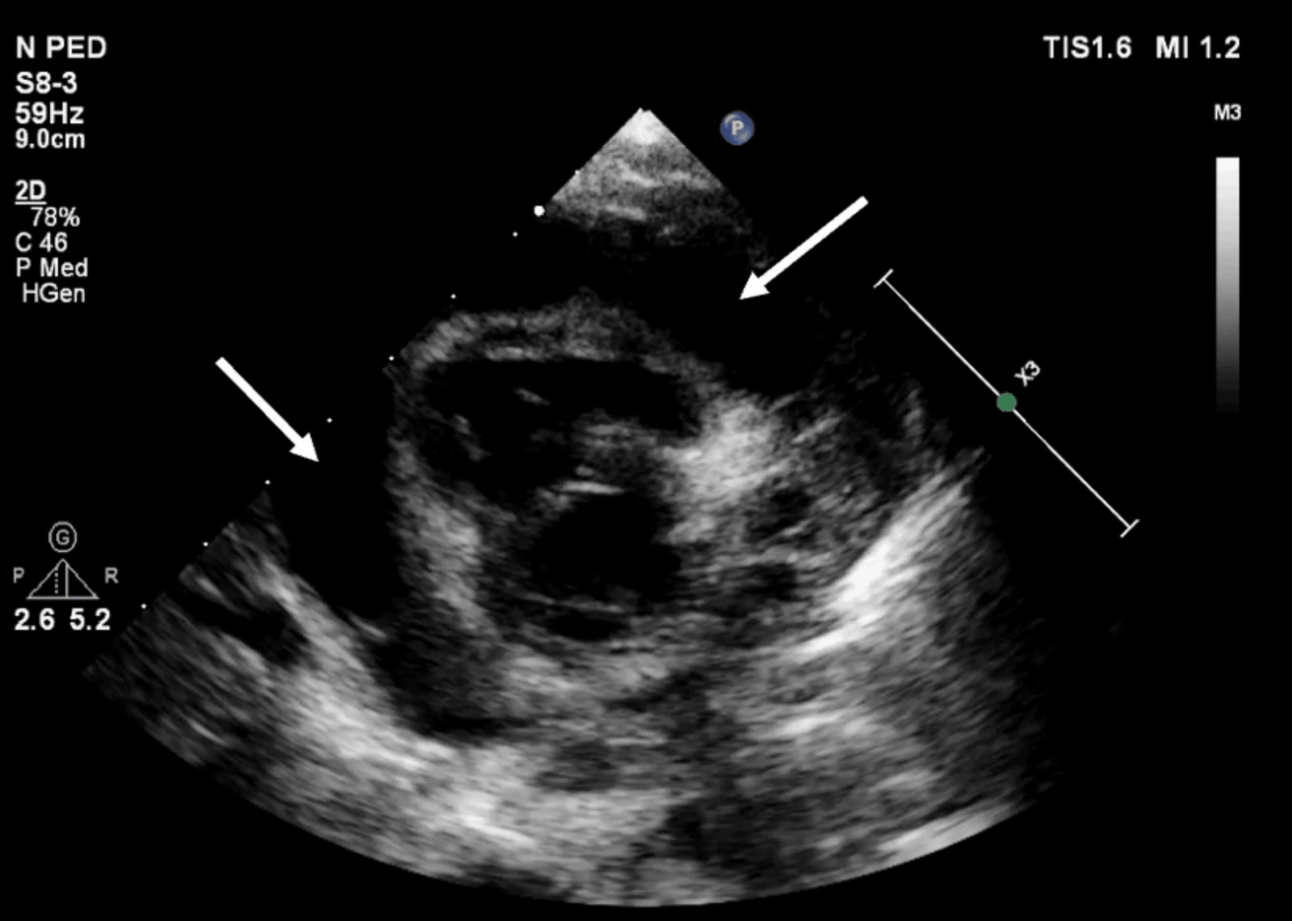
Transthoracic echocardiography (TTE) parasternal short axis 2D view showing circumferential large pericardial effusion (at the time of presentation)

Echocardiography (Figures 2, 3) revealed a large pericardial effusion and left ventricular ejection fraction of 20% but no signs of cardiac tamponade.

**Figure 4:**
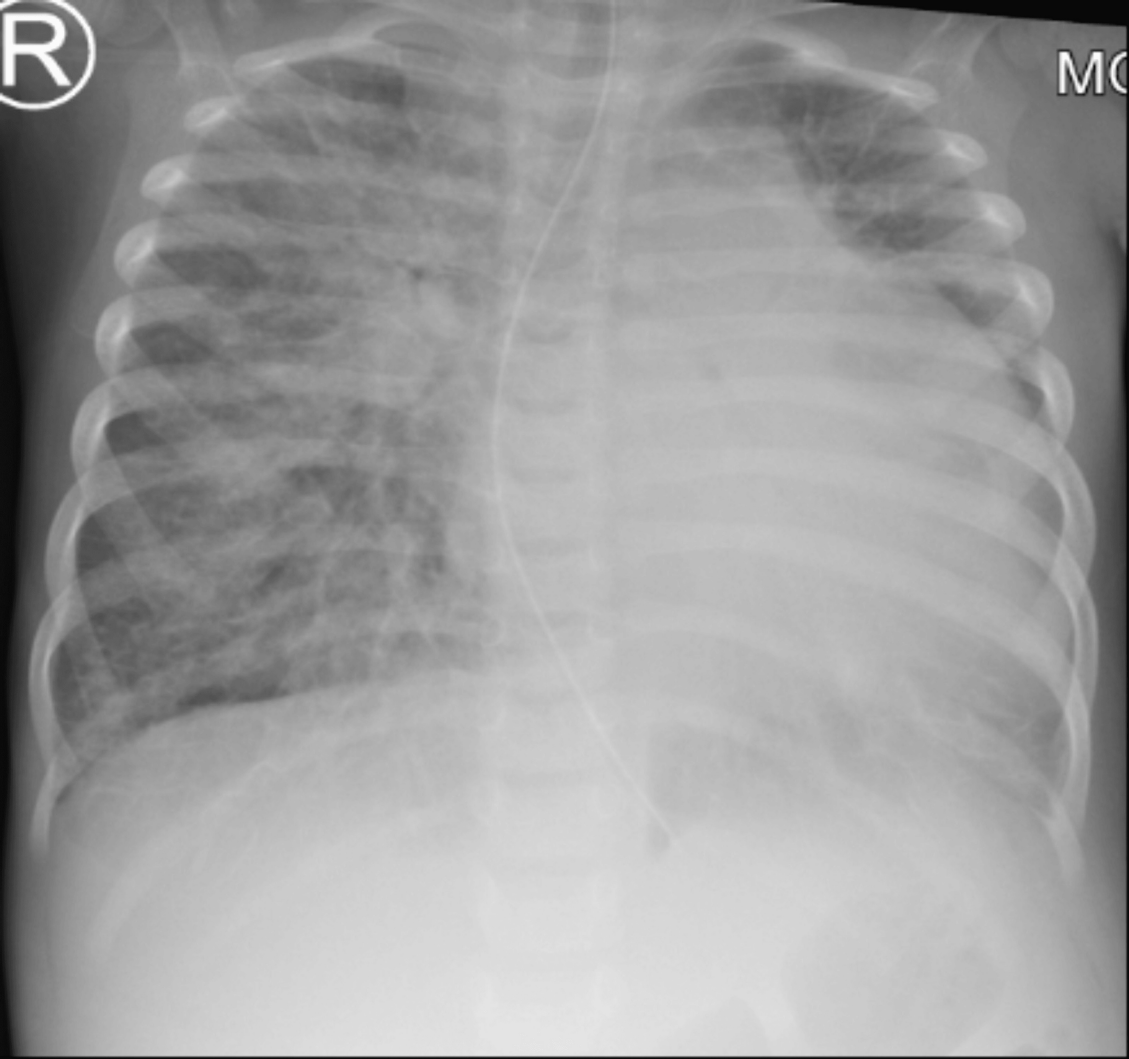
Chest X-ray at day five of admission shows cardiomegaly and pulmonary edema

On initial investigation, she had anemia (Haemoglobin 8.6 g/dL, reference: 11.5 - 15.5), proteinuria (nephrotic range), and hypoalbuminemia, but her kidney function was normal. She was admitted to the pediatric intensive care unit with nephritic-nephrotic picture with ascites, hypertension, and pericardial effusions that were thought to be secondary to SLE. For respiratory distress, she received high-flow nasal cannula, amlodipine for hypertension, diuretics with albumin to treat oedema, ascites and pericardial effusion. She was started on methylprednisolone pulse therapy (30 mg/kg once a day), mycophenolate mofetil (25 mg/kg twice a day) and hydroxychloroquine (5 mg/kg once a day). She developed cardiogenic shock on day five of admission and required intubation and inotropic support. Repeated chest X-ray showed cardiomegaly and pulmonary edema (Figure 4).

She developed acute kidney injury secondary to shock which necessitated peritoneal dialysis for a few days and had full recovery of renal function. There were no signs of cardiac tamponade and she did not require pericardiocentesis. Due to the significant involvement of both the cardiac and renal systems, she was given intravenous immunoglobulin (IVIG at 1 gram/kg). Mycophenolate was discontinued and replaced with cyclophosphamide pulse therapy (20 mg/kg) administered every two weeks for six doses as an induction therapy. Her heart function and pericardial effusion improved gradually, and she was extubated on day 10. Following this, mycophenolate mofetil (25 mg/kg twice a day) was reintroduced for maintenance therapy, along with oral prednisolone (0.5 mg/kg once a day) and hydroxychloroquine (10 mg/kg once a day). Further investigations confirmed the diagnosis of SLE, with positive anti-nuclear antibodies (ANA) and anti-double-stranded DNA (dsDNA) antibodies and low C3 and C4 complement as shown in Table 1.

**Table 1:** Results of the investigations ESR: Erythrocyte sedimentation rate, CRP: C-reactive protein

Investigation	Result	Reference
Anti-nuclear antibodies (ANA)	Reactive, 1:320	Cut-off 1:100
Anti-ds DNA antibodies	347.1 IU/mL	Positive >100, Negative <100
C3 complement	124 mg/L	800-1500
C4 complement	8 mg/L	120-360
Urine protein creatinine ratio	761.9 mg/mmol	Normal <20, Nephrotic >200
Lupus anticoagulant screen	Negative	-
C1Q antibodies	Negative	Negative <10
ESR	41 mm/h	2-30
CRP	<4 mg/l	<10
Albumin	21 g/l	34-50

**Figure 5:**
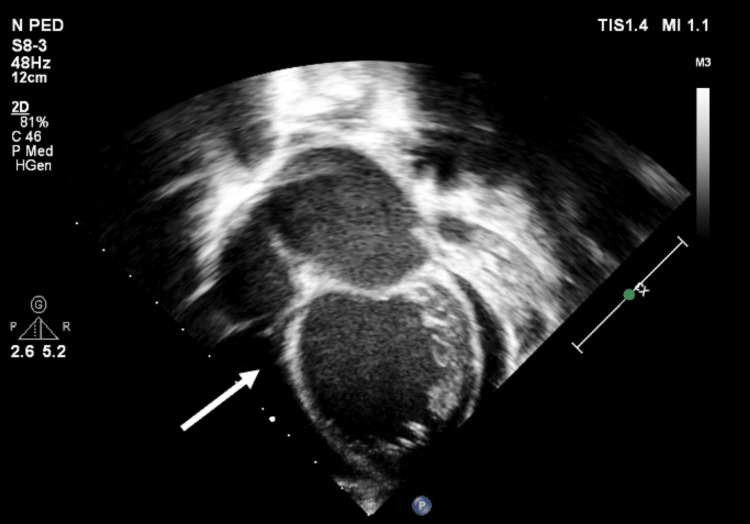
Follow-up echocardiography after six months showing minimal pericardial effusion (apical four chamber view)

**Figure 6:**
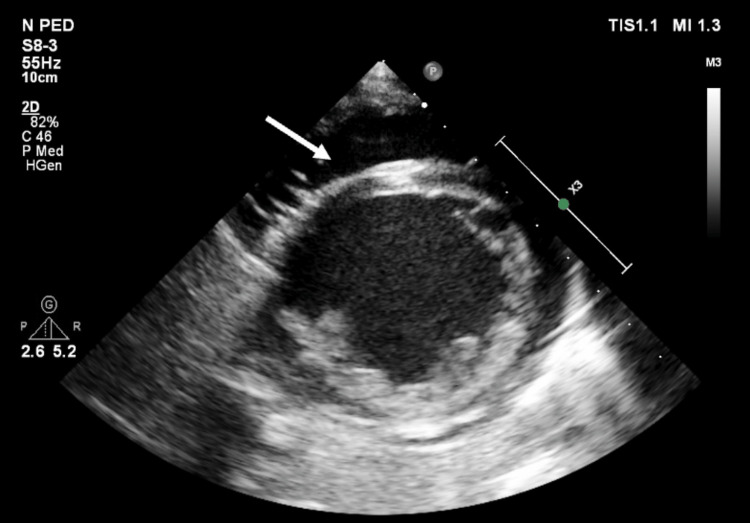
Follow-up echocardiography after six months showing minimal pericardial effusion (parasternal short axis 2D view)

Whole exome sequencing revealed a homozygous pathogenic variant (c.643del p. (Trp215Glyfs*2) in the DNASE1L3 gene, indicating autosomal recessive systemic lupus erythematosus type 16. A six-month follow-up revealed minimal pericardial effusion and a 56% ejection fraction (Figures 5, 6).

## Discussion

This report describes a 22-month-old infant, where pericardial effusion emerged as the first manifestation of the disease, which has not been previously reported in children under the age of three. SLE is a complex autoimmune disorder that affects multiple organ systems, including the heart, kidneys, and lungs [3]. cSLE presents unique challenges, especially in younger patients. The term cSLE refers to those who develop SLE before the age of 18 years old. The median age at which cSLE occurs is 11-12 years, with more severe disease at presentation, and it rarely affects children under five [9,10]. Compared to adult-onset SLE, cSLE has a higher prevalence of initial and cumulative multiorgan system involvement [11]. Based on the data of 141 pediatric SLE patients, the median age at diagnosis was 10.8 years, and fever, vasculitis rash, and lethargy were the most commonly reported clinical features during presentation [12]. Cardiac involvement is one of the most severe clinical manifestations of SLE, which is associated with significant morbidity and mortality [13]. It includes pericarditis, myocarditis, endocarditis, pericardial effusions, and cardiac tamponade [14]. Compared to adults, children with SLE had a significantly higher incidence of pericarditis and myocarditis in the first year of diagnosis compared to adults but fewer valvular diseases [4]. A study of 104 children with cSLE in Oman found that 16% of patients had cardiac manifestations, with a mean age of eight years but it did not elaborate on the types of cardiac involvements [15]. According to a study in the United States involving 297 children with SLE, pericarditis accounted for 10.4% of cardiac manifestations, followed by valvular insufficiency (9.1%), and myocarditis and endocarditis were less common (1.0% and 1.0%, respectively) [4]. In the same study, two children only (0.67%) had cardiac tamponade that required pericardiac drainage and it was found that cardiac manifestations were evident within a year after diagnosis, but not on presentation. As a first presentation of SLE, pericardial effusion and tamponade were only reported in case reports in adults and adolescents, but never in children under three years old [5-8]. According to our knowledge and literature review, our patient is the youngest infant to experience pericardial effusion as a first sign of SLE. In 2010, a three-year-old child was reported with pericardial effusion complicated by cardiac tamponade as the first presentation of SLE [6]. Other pediatric cases with cardiac manifestations as an initial presentation of SLE are shown in Table 2.

**Table 2:** Reported pediatric systemic lupus erythematosus (SLE) cases with cardiac involvement as a first presentation

No	Study	Year of publication	age (years)	Gender	Cardiac manifestation
1	Arabi MT. et al. [5]	2012	9	M	Cardiac tamponade
2	Arabi MT. et al. [5]	2012	11	F	Cardiac tamponade
3	Ulas Saz. et al. [6]	2010	3	F	Cardiac tamponade
4	Chen YJ. et al. [7]	2022	11	F	Myocarditis and pericardial effusion
5	Huang CN. et al. [8]	2013	12	F	Myocarditis and pericardial effusion

Pericardial effusion is a life-threatening condition that can lead to cardiac tamponade. An extensive workup is needed for children with pericardial effusion to rule out infectious and non-infectious causes, including autoimmune diseases such as SLE [16]. SLE cardiac involvement may be treated with steroids, cyclophosphamide, and IVIG [8,17-19]. The management of such patients requires a multidisciplinary approach, as demonstrated in this case where multiple teams were involved including intensivist, cardiologist, rheumatologist and nephrologist. The combination of methylprednisolone pulse therapy, mycophenolate mofetil, hydroxychloroquine, and IVIG aimed at controlling inflammation and modulating the immune response was essential in stabilizing the patient. As the patient’s condition progressed, the addition of cyclophosphamide pulse therapy and the adjustment of immunosuppressive treatment were necessary due to the extent of the renal and cardiac involvement. The evidence around therapeutic options is beyond the scope of this case report. The genetic finding highlights the importance of genetic screening in pediatric cases of SLE, especially when atypical presentations, such as early-onset pericardial effusion, are observed. The identification of genetic mutations in DNASE1L3 provides valuable insights into the pathogenesis of SLE, as DNASE1L3 is involved in the regulation of DNA breakdown, and mutations in this gene are associated with increased susceptibility to autoimmune diseases like SLE.

## Conclusions

This case highlights the importance of suspecting SLE and considering it in the differential diagnosis of children with pericardial effusion. Early immunosuppressive therapy, combined with supportive care, can significantly improve outcomes in these patients. Additionally, genetic testing plays a crucial role in confirming the diagnosis and understanding the underlying etiology of rare, early-onset forms of SLE. Future studies are needed to explore the long-term outcomes of children with genetic mutations associated with SLE and to establish standardized treatment protocols for this vulnerable population.
